# Glatiramer acetate treatment persistence - but not adherence - in multiple sclerosis patients is predicted by health-related quality of life and self-efficacy: a prospective web-based patient-centred study (CAIR study)

**DOI:** 10.1186/s12955-017-0622-z

**Published:** 2017-03-14

**Authors:** Peter Joseph Jongen, Wim A. Lemmens, Erwin L. Hoogervorst, Rogier Donders

**Affiliations:** 1 University Medical Centre Groningen, Department of Community and Occupational Medicine, University Groningen, Antonius Deusinglaan 1, 9713 AV Groningen, The Netherlands; 2 MS4 Research Institute, Ubbergseweg 34, 6522 KJ Nijmegen, The Netherlands; 3 Department for Health Evidence, Radboud University Medical Centre, P.O. Box 9101, 6500 HB Nijmegen, The Netherlands; 4 St. Antonius Hospital, P.O. Box 2500, 3430 EM Nieuwegein, The Netherlands

**Keywords:** Multiple sclerosis, Relapsing remitting, Persistence, Adherence, Self-efficacy, Quality of life, Health-related quality of life, Glatiramer acetate, Disease modifying treatment

## Abstract

**Background:**

In patients with relapsing remitting multiple sclerosis (RRMS) the persistence of and adherence to disease modifying drug (DMD) treatment is inadequate. To take individualised measures there is a need to identify patients with a high risk of non-persistence or non-adherence. As patient-related factors have a major influence on persistence and adherence, we investigated whether health-related quality of life (HRQoL) and self-efficacy could predict persistence or adherence.

**Methods:**

In a prospective web-based patient-centred study in 203 RRMS patients, starting treatment with glatiramer acatete (GA) 20 mg subcutaneously daily, we measured physical and mental HRQoL (Multiple Sclerosis Quality of Life-54 questionnaire), functional and control self-efficacy (Multiple Sclerosis Self-Efficacy Scale), the 12-month persistence rate and, in persistent patients, the percentage of missed doses. HRQoL and self-efficacy were compared between persistent and non-persistent patients, and between adherent and non-adherent patients. Logistic regression analysis was used to assess whether persistence and adherence were explained by HRQoL and self-efficacy.

**Results:**

Persistent patients had higher baseline physical (mean 58.1 [standard deviation, SD] 16.9) and mental HRQoL (63.8 [16.8]) than non-persistent patients (49.5 [17.6]; 55.9 [20.4]) (*P* = 0.001; *P* = 0.003) with no differences between adherent and non-adherent patients (*P* = 0.46; *P* = 0.54). Likewise, in persistent patients function (752 [156]) and control self-efficacy (568 [178]) were higher than in non-persistent patients (689 [173]; 491 [192]) (*P* = 0.009; *P* = 0.004), but not in adherent vs. non-adherent patients (*P* = 0.26; *P* = 0.82). Logistic regression modelling identified physical HRQoL and control self-efficacy as factors that explained persistence. Based on predicted scores from the model, patients were classified into quartiles and the percentage of non-persistent patients per quartile was calculated: non-persistence in the highest quartile was 23.4 vs. 53.2% in the lowest quartile. Risk differentiation with respect to adherence was not possible. Based on these findings we propose a practical work-up scheme to identify patients with a high risk of non-persistence and to identify persistence-related factors.

**Conclusions:**

Findings suggest that pre-treatment physical HRQoL and control self-efficacy may identify RRMS patients with a high risk of early discontinuation of injectable DMD treatment. Targeting of high-risk patients may enable the efficient use of persistence-promoting measures.

**Trial Registration:**

Nederlands Trial Register code: NTR2432.

**Electronic supplementary material:**

The online version of this article (doi:10.1186/s12955-017-0622-z) contains supplementary material, which is available to authorized users.

## Background

In patients with relapsing remitting multiple sclerosis (RRMS) treatment with disease modifying drugs (DMDs) is associated with a decrease in the frequency and severity of relapses [1], and a delayed conversion to secondary progression [2]. In general, high DMD exposure is associated with better clinical outcomes than low exposure [3], and a continued use is thought to provide the greatest long term benefit [4, 5]. However, medication for chronic illness is only taken by 50 – 60% of the patients as prescribed [6, 7]. Not taking medication as agreed upon includes two different patient behaviours: missing doses (non-adherence) and premature treatment discontinuation (non-persistence) [7]. According to a World Health Organization report five interacting dimensions affect adherence and persistence: social and economic factors, health care team and system-related factors, condition-related factors, therapy-related factors and patient-related factors. The latter factors represent the resources, knowledge, attitudes, beliefs, expectations and perceptions of the patient [7].

Health-related quality of life (HRQoL) is a multi-dimensional concept that focuses on the impact health status has on physical, mental, emotional and social dimensions of quality of life [8]. As an overall measure from a patient’s perspective, HRQoL measures the disease impact on health dimensions that can not be evaluated using observer-based measures of physical disability [9–11]. In a web-based survey among MS patients, higher HRQoL was associated with better adherence to injectable DMD treatment [12]. In a previous study of HRQoL in RRMS patients starting IM interferon-beta (INFb) we found that patients who discontinued treatment within 24 months had a lower physical or mental HRQoL at baseline than those who continued treatment [13]. Theoretically, as an overall patient-centerd concept, HRQoL may be thought to comprehensively reflect patient-related factors affecting persistence and adherence.

Self-efficacy is a psychological concept that refers to the degree in which a person is confident to complete tasks and reach goals in specific situations [14]. It is a core component in social cognitive theory, in which psychosocial functioning is determined by reciprocal interactions between personal factors, behaviour and the environment [15, 16]. In MS patients low self-efficacy has been associated with less psychological adjustment [17]. Two retrospective studies in the U.S.A. among patients treated with glatiramer acetate (GA) 20 mg subcutaneously (sc) daily identified self-efficacy as predictor of persistence, along with hope and perception of support, whereas in a prospective study in GA-treated patients self-efficacy was significantly related to 3-month persistence [18–20].

Discontinuation of injectable DMD treatment in RRMS patients mostly occurs in the first 12 months [21–23]. After 6 months up to 27% may have discontinued treatment [21], and after 14 months 43% of those initiating therapy reported to be non-persistent [22].

From a practical point of view it could be useful to neurologists and nurses to be able to identify those patients who are at high risk of early treatment discontinuation or missing doses. Although various patient-related factors have been found to be associated with non-persistence or non-adherence, to our knowledge no study provided an algorithm that quantifies the risk of inadequate drug us in individual patients. In view of the study reports and considerations mentioned above we investigated in RRMS patients starting treatment with GA 20 mg sc daily, whether persistence and adherence were associated with HRQoL and self-efficacy, and whether eventual associations could result in the development of a predictive model for use in daily clinical practice [24]. So, we analyzed self-efficacy and HRQol in persistence vs. non-persistent, and in adherent vs. non-adherent patients, and used logistic regression modelling to predict persistence and adherence with HRQoL and self-efficacy as independent variables.

## Methods

### Study design

The present analysis is part of the CAIR study, an investigator-initiated, prospective, web-based, patient-centred, observational study in The Netherlands. The study’s name is an acronym from the principal analytical method, main theme and study population: Correlative analysis of Adherence In Relapsing remitting MS (CAIR). The study duration was 12 months. Nederlands Trial Register code: NTR2432. GA 20 mg sc daily was prescribed by neurologists as per regular care, dispensed as a commercial drug by general pharmacies (Copaxone ®) and administered by the patient according to the instructions in the package leaflet.

The primary outcomes were the relationships between adherence and persistence, and the numbers of care sessions and the quantity of care per discipline. The secondary outcomes were the relationships between adherence and persistence, and patient characteristics, socio-economic situation, health care, caregivers, disease and treatment [24].

Patients were informed by neurologists, MS-nurses and specialised nurses who teach patients to self-inject, and via websites of patient organisations. Patients were also advised to visit the study website. For further information they could contact the study helpdesk by telephone or e-mail, or the coordinating investigator (PJJ) by e-mail. The recruitment period was from July 2009 to July 2011 and comprised two phases. First, in 2009 15 MS-specialized neurological practices with an MS-nurse were recruited as study sites. The practices were fairly distributed over the country. Since July 2009 they informed patients starting GA treatment of the possibility to participate in the study. Objectives and overall requirements were discussed with the patient. The study information was preceded by the decision to start GA treatment. When a patient decided to participate, the neurologist or the MS-nurse notified the study helpdesk and participation was started. Second, since February 2010 nurses who teach patients to self-inject GA briefly informed patients about the study. Patients interested in receiving further information were handed a postage paid card addressed to the study helpdesk. On receipt of the card the helpdesk contacted the patient by phone and provided information. Patients who, after being informed, were willing to participate either signed the informed consent form at their neurologist’s or MS-nurse’s office or, in case the neurologist was not yet involved in the study, confirmed the text of the informed consent by clicking on a specific page of the study website. In the latter case the coordinating investigator contacted the neurologist by phone or e-mail to introduce the study, and provided the study protocol with informed consent text. Within 2 weeks a second contact was established and the neurologist informed the coordinating investigator on his/her decision to participate or not. If the neurologist participated he/she and the MS-nurse were contacted by the helpdesk and the practice was activated as study site. A negative decision by the neurologist did not interfere with the patient’s participation, as the study was patient-centred and the primary research question could be answered by patient-derived data only.

The inclusion criteria were 1) indication for GA treatment, 2) being relapse free and having stable symptoms for at least 30 days, 3) willing and able to comply with the protocol for the duration of the study, and 4) having given written informed consent. The exclusion criteria were 1) contra-indication to GA as defined in the Summary of Product Characteristics text, 2) hypersensitivity to GA or mannitol, 3) worsening of symptoms suggestive of a relapse, 4) pregnancy or lactation, and 5) the time interval between the first GA injection and baseline assessment was more than 4 weeks.

### Technical aspects

The study was a modular application on the Curavista e-health platform, built on an Oracle database with JAVA-scripting, XML-applets and AJAX protocols. Data processing was 256-bits encrypted with VPN-tunnelling. The databases were physically and software secured in a dedicated data centre in The Netherlands. The database of the study was compliant with EU-regulations on data storage and activation for medical purposes. There were four separated databases: one with personal identifiers (name, address, identification number), one with study records (answers to the questions, identification number), one with the social security number, and one with the key. Only after login the data were presented as a whole on the screen (encrypted key).

### Data acquisition

Data were acquired via a special study website. Patients logged in with a code provided by the study help desk and chose a username and password. Online they went through web pages containing the electronic case record forms (eCRFs). The questions and questionnaires related to missed doses, discontinuation, adverse events, medication, relapses, self-efficacy, HRQoL and other adherence- or persistence-related factors [24].

Patients were informed by email that an assessment was due and that the corresponding forms had been made available for completion. eCRFs were to be completed within one week. Completion could take as many sessions as needed, as answers were saved automatically. After confirmation by the patient the eCRF was automatically sent to the study centre. Incomplete eCRFs were returned. In case an eCRF was not completed within one week, the help desk reminded the patient by telephone.

### Outcome measures

HRQoL was assessed via the Multiple Sclerosis Quality of Life 54-Items (MSQoL-54) questionnaire [25]. The MSQoL-54 is a psychometrically validated, MS-specific, multi-dimensional inventory of patient-centered health status, and consists of the Short Form 36-Item health survey as a generic core measure, supplemented with 18 questions on items relevant to persons with MS in the areas of health distress, sexual function, satisfaction with sexual function, overall quality of life, cognitive function, energy, pain and social function [25]. The MSQoL-54 contains 52 items distributed into 12 scales, and two single items. A physical and a mental dimension underlie the MSQoL-54: the Physical and Mental domains [25]. Scores for each domain range from 0 to 100, where higher values indicate better HRQoL.

Self-efficacy was assessed by means of the Multiple Sclerosis Self-Efficacy Scale (MSSES). The MSSES is an 18-item, psychometrically validated, self-report questionnaire for the assessment of self-efficacy in patients with MS [26]. The MSSES consists of two 9-item subscales of Function and Control. Each item is scored on a Likert-like scale form 10 (very uncertain) to 100 (very certain) and addition of the respective item scores yields the MSSES Function score and the MSSES Control score, both ranging from 90 (minimum) to 900 (maximum). The MSSES Function subscale measures confidence with functional abilities, whereas the MSSES Control subscale measures confidence with managing symptoms and coping with the demands of the illness [26]. The completion of the HRQoL and self-efficacy questionnaires took about 20 to 30 min.

The number of missed GA doses in the preceding 14 days and eventual GA discontinuation, whichever was applicable, were recorded by patients. The completion of the questionnaire about missed GA doses (adherence) and treatment (dis) continuation (persistence) took less than 5 min.

### Assessment schedule

The MSQoL-54 and MSSES questionnaires were completed at baseline. Adherence and persistence were assessed at six predefined and six random time points, the latter being also unknown to neurologists and MS-nurses; thus, adherence and persistence were assessed at 4, 10, 12, 16, 20, 26, 32, 34, 38, 44, 48 and 52 weeks.

### Statistical analyses

In a previous CAIR study report on the relationship between persistence and adherence, and the quantity of care received from multiple disciplines, we found that 95% injected doses was the preferred cut-off point for adherence. For, the application in persistent patients of 15, 10, 5 and 1% missed doses as cut-off points resulted in 85, 90, 95 and 99% adherence rates in 99.19, 92.74 84.68 and 47.58% of the patients, respectively; to maximize our chances to find statistically significant and clinically relevant differences between adherent and non-adherent patients we chose 95% adherence as the cut-off point for non-adherence.

The mean, standard deviation (SD), minimum and maximum values of the baseline MSQoL-54 Physical, MSQoL-54 Mental, MSSES Function and MSSES Control scores were calculated in non-persistent, persistent, persistent non-adherent, and persistent adherent patients. Differences between non-persistent and persistent patients, and between persistent non-adherent and persistent adherent patients were tested by means of Student’s *t*-test using 0.05 as level of significance.

To assess the extent to which persistence and adherence could be explained by the MSQoL-54 Physical, MSQoL-54 Mental, MSSES Function and MSSES Control scores at baseline, we performed a stepwise logistic regression analysis with forward selection using one or more of the variables. The order of entering the variables into the model was determined by the *P* values of the correlation with the outcome variable.

## Results

### Patients, persistence and adherence

Two-hundred-and-three patients were included in the study, three of which did not complete a single questionnaire [24]. Of the resulting 200 analyzable patients, the female-to-male ratio was 3.65:1, the mean age 39.7 years (SD 9.8, minimum 19, maximum 62), and the mean disease duration (*N* = 107) 4.5 years (SD 5.0, minimum 0, maximum 18) [24]. One-hundred-and-twenty-four (62%) patients were treatment persistent as they continued GA injections throughout the 12-month study period. In the persistent group 105 (84.7%) patients were 95% adherent. Treatment persistence was not related to sex (*Χ* ^2^ = 0.89, *P* = 0.35) nor to age (*T*-test = −0.52, *P* = 0.60), neither was treatment adherence related to sex (*Χ* ^2^ = 0.04, *P* = 0.84) or to age (*T*-test = −0.50, *P* = 0.60).

### MSQoL-54 and MSSES scores

The mean, SD, minimum and maximum values of the MSQoL-54 Physical and Mental scores at baseline in non-persistent, persistent, persistent non-adherent, and persistent adherent patients are presented in Table 1. The persistent group had higher MSQoL-54 Physical scores than the non-persistent group (*T*-test = −3.34, *P* = 0.001), whereas between the non-adherent and adherent persistent patient groups the MSQoL-54 Physical scores did not differ (*T*-test = −0.73, *P* = 0.46). Similarly, the MSQoL-54 Mental scores were higher in persistent than in non-persistent patients (*T*-test = −2.96, *P* = 0.003), but there was no difference between persistent non-adherent and persistent adherent patients (*T*-test = −0.62, *P* = 0.54).

**Table 1:** MSQoL-54 Physical and Mental scores in non-persistent, persistent, persistent non-adherent, and persistent adherent patients at baseline

	MSQoL-54 Physical			
	Non-persistent (*n* = 70)	Persistent (*n* = 119)	Persistent non-adherent (*n* = 18)	Persistent adherent (*n* = 101)
Mean (SD) (min.-max.)	49.5 (17.6)* (13.0-91.5)	58.1 (16.9)* (21.3-94.8)	55.5 (15.2) (29.6-79.4)	58.6 (17.2) (21.3-94.8)
	MSQoL-54 Mental			
	Non-persistent (*n* = 74)	Persistent (*n* = 123)	Persistent non-adherent (*n* = 19)	Persistent adherent (*n* = 104)
Mean (SD) (min.-max.)	55.9 (20.4)^#^ (21.2-94.4)	63.8 (16.8)^#^ (19.8-90.6)	61.7 (14.9) (33.5-83.4)	64.2 (17.2) (19.8-90.6)

*MSQoL-54*, Multiple Sclerosis Quality of Life-54, *min*. minimum; *max*. maximum

*P = 0.001; ^#^ *P* = 0.003

The mean, SD, minimum and maximum values of the MSSES Function and Control scores at baseline in non-persistent, persistent, persistent non-adherent, and persistent adherent patients are presented in Table 2. The persistent group had higher MSSES Control scores than the non-persistent group (*T*-test = −2.89, *P* = 0.0042). Between the persistent non-adherent and persistent adherent groups the MSSES Control scores did not differ (*T*-test = 0.23, *P* = 0.82). Likewise, the persistent group had higher MSSES Function scores than the non-persistent group (*T*-test = −2.65, *P* = 0.0087), whereas the MSSES Function scores did not differ between persistent non-adherent and persistent adherent patient groups (*T*-test = −1.12, *P* = 0.26).

**Table 2:** MSSES Function and Control scores in non-persistent, persistent, persistent non-adherent, and persistent adherent patients at baseline

	MSSES Function			
	Non-persistent (*n* = 58)	Persistent (*n* = 131)	Persistent non-adherent (*n* = 19)	Persistent adherent (*n* = 112)
Mean (SD) (min.-max.)	689 (173)* (280–900)	752 (156)* (90–900)	715 (182) (240–900)	758 (151) (90–900)
	MSSES Control			
	Non-persistent (*n* = 58)	Persistent (*n* = 131)	Persistent and non-adherent (*n* = 19)	Persistent and adherent (*n* = 112)
Mean (SD) (min.-max.)	491 (192)^#^ (130–900)	568 (178)^#^ (90–900)	560 (140) (210–780)	570 (185) (90–900)

*MSSES* Multiple Sclerosis Self-Efficacy Scale, *min*. minimum, *max*., maximum

*p = 0.0087, ^#^ *p* = 0.0042

### Logistic regression analyses

Regarding treatment persistence, at the first step the MSQoL-54 Physical score entered the model (*P* = 0.001) (MSSES Control *P* = 0.0031, MSQoL-54 Mental *P* = 0.0089, MSSES Function *P* = 0.0134). At the second step the MSSES Control score was entered (*P* = 0.2629) (MSQoL-54 Mental *P* = 0.5777, MSSES Function *P* = 0.5395). Then, based on the predicted scores resulting from the logistic regression model, we classified individual patients into quartiles and calculated the percentage of non-persistent patients per quartile. The overall percentage of non-persistence in the group (*N* = 189) was 37% (see above). Whereas the percentage of non-persistent patients in the highest quartile was 23%, in the lowest quartile it was 53%, viz. 2.27 times higher (Table 3).

**Table 3:** Percentage and number of persistent vs. non-persistent patients per quartile of predicted scores based on baseline MSQoL-54 Physical and MSSES Control scores

Quartile	Percentage non-persistent patients	Percentage persistent patients	*N* total
4	23.40 (*n* = 11)	76.60 (*n* = 36)	47
3	25.00 (*n* = 12)	75.00 (*n* = 36)	48
2	46.81 (*n* = 22)	53.19 (*n* = 25)	47
1	53.19 (*n* = 25)	46.81 (*n* = 22)	47
Total	37.04 (*n* = 70)	62.96 (*n* = 119)	189

*MSQoL*-54 Multiple Sclerosis Quality of Life-54, *MSSES* Multiple Sclerosis Self-Efficacy Scale; *Χ* ^2^ = 13.91, *p* = 0.0030

The point estimate of the MSQoL-54 Physical effect was 1.022, and the point estimate of the MSSES Control effect was 1.011. In other words, one point increase in the MSQoL-54 Physical score increases the predicted score by 2.2%, and ten points increase in the MSSES control score increases the predicted score by 1.1%.

With respect to adherence, not a single score entered the model (MSSES function *P* = 0.2340, MSQoL-54 Physical *P* = 0.4607, MSQoL-54 Mental *P* = 0.7891, MSSES Control *P* = 0.8207).

## Discussion

In a prospective 12-month study in RRMS patients starting treatment with GA 20 mg sc daily we found, first, that treatment persistence – but not adherence - was positively associated with HRQoL and self-efficacy at baseline, and, second, that a predictive model based on these two measures might differentiate between patients with a low risk (about 23%) vs. those with a high risk (about 53%) of treatment discontinuation within 12 months.

In the treatment of chronic disorders patient-related factors are crucial to persistence and adherence [7]. HRQoL is a multi-dimensional concept that covers the impact disease has on the patient’s physical, mental, emotional and social well-being. Since HRQoL is an overall measure from a patient’s perspective, we hypothesized that in MS patients pre-treatment HRQoL might relate to persistence of and adherence to DMD treatment. It was found that physical HRQoL seemed to predict 12-month persistence. To understand this relationship it is of note that in MS patients the discontinuation of DMD treatment has been shown to be significantly influenced by the disability level [27]. In fact, RRMS patients with high disease activity and high disability are likely to benefit less from first-line DMD treatments, and may therefore be at a higher risk of early discontinuation. It has also been known that in MS patients physical HRQoL correlates with the severity of the disease, e.g. as measured by the physician-reported Expanded Disability Status Scale and the relapse rate [9, 28]. Thus, there is a link to be conceived between pre-treatment low physical HRQoL and early treatment discontinuation. If this relationship is substantial, then it would also apply to drug treatments of other chronic disorders. Actually, in a large naturalistic study in patients with schizophrenia the 12-month treatment completion with olanzapine was related to a better HRQoL at baseline [29].

In our study the adherence to the dosing schedule was not related to pre-treatment HRQoL. This seems to contrast with an international cross-sectional study in over 1900 RRMS patients, finding that adherent patients had a better quality of life - also with respect to physical well-being and symptoms - than non-adherent patients [27]; in addition, women were more likely than men to adhere to treatment [27]. It has also been demonstrated that patients with low disability – and thus a conceivably higher HRQoL - do miss less doses [30]. The fact that our findings are not in line with these reports may result from the smaller sample size of our study, our definition of non-adherence, or - more likely - to us relating adherence to the *pre-treatment* HRQoL. As we reported previously, HRQoL may increase substantially within 3 months after start of GA treatment and remain so for at least 24 months; so, it may well be that the increase in HRQoL after the start of treatment explains for the relationship between *on-treatment* HRQoL and adherence, as better adherence will expectedly result in a higher increase in HRQoL [31, 32].

Our data suggest that adherence is to a certain degree influenced by other factors than those affecting persistence, the latter being lack of effectiveness and side effects. A study in hypertensive patients indicated that events interfering with daily routine had a significant impact on adherence, and that adherence appears to be a patterned behaviour established through the creation of a routine and a reminder system for taking the medication [33]. Recently, it has been found that in GA-treated MS patients the most common reason for missing injections is that they simply forget to administer the medication [12], other factors being not feeling like taking or being tired of taking the injections, skin reactions, pain at injection sites, injection-related anxiety and the absence of someone to help administer the medication [12]. Similarly, non-adherence among kidney transplant patients on immunosuppressant therapy, where admitted, was unintentional [34] and due to forgetfulness, interference with lifestyle, change in routine, and impact of side effects [34]. So, it is likely that in patients with chronic disorders non-adherence primarily relates to cognitive, practical and treatment-related factors, which are in part different from those affecting persistence and are, except from cognitive factors, not reflected by pre-treatment HRQoL.

Another finding in our study was that control self-efficacy seemed to predict 12-month persistence. Control self-efficacy reflects the individual’s sense of confidence that he/she can control disease symptoms, reactions to disease-related limitations, and the effect of the disease on life activities [26]. Thus, the ‘control self-efficacy’ concept links the patient’s self-confidence to his/her disease state. Already in 2001 Fraser et al., measuring self-efficacy via the MSSES in a retrospective survey in 341 GA-treated RRMS patients, found that those who had not discontinued treatment in the first year, had a higher level of control self-efficacy (mean 595, SD 184) than the patients who had discontinued (mean 532, SD 182) [18]; in contrast to our study, the function self-efficacy score did not differ between persistent en non-persistent patients (mean 742, SD 166 vs. mean 712, SD 167) [18]. In a prospective study Zwibel et al. found that greater function self-efficacy predicted 12-week GA persistence in treatment-naive patients, but not in treatment-experienced patients [20].

Cognitive problems were not an exclusion criterion for study participation, in spite of their frequent occurrence in MS [35]. This choice was motivated by the following considerations. First, given the real-life nature of the study, exclusion of cognitively impaired patients would have interfered with the generalisability of the study conclusions. Second, it has been shown that cognitive impairment in MS does not affect reliability and validity of self-report health measures, including HRQoL [36].

Greater use of screening and assessment tools is needed to identify and target the patients who are at the greatest risk for not taking medication as agreed [37]. And although we know about common features of adherence or persistence improving programs, it is difficult to determine the best possible combination of such features for any given patient [37]. Zwibel et al. suggested that findings in GA-treated RRMS patients on the relationship between HRQoL/self-efficacy and persistence may justify interventions to improve persistence [20]. Based on our observations, we propose a prognostic and interventional work-up scheme to identify those RRMS patients starting a first-line injectable DMD treatment who are at high risk (>50%) of early discontinuation, and to guide in these patients the use of persistence improving measures (Table 4). In Step 1 physical HRQoL and control self-efficacy are assessed and a predictive score is calculated to identify high risk patients. Once the right patients are identified, interventions can be tailored to individual patients [37]. In our proposal step 2A focuses on the screening of symptoms that are known to be associated with a lower HRQoL in MS: depression, anxiety, fatigue and bladder symptoms; it had already been recognized that treatment guidelines for chronic conditions should recommend screening for depression, as this can be an indicator of poor persistence and adherence [37, 38]. Then, to improve the chances of persistence in individual patients, we propose interventional measures to be taken based on abnormal outcomes of the additional screenings. In step 2B we suggest to re-assess prognostic factors in order to detect patients with an expectedly unfavourable disease course, in whom a first-line DMD may be insufficiently effective. Interestingly, a recent study, showing that in MS patients depression, anxiety, and fatigue are associated with a decreased HRQoL, is in line with our approach [39]. It is of note that the idea to address multiple HRQoL-related factors presupposes, first, that these factors relate causally to persistence and, second, that positively influencing these factors will improve persistence. The concept of addressing multiple HRQoL-related factors simultaneously is supported by a recent review, which states that multi-component interventions showed the strongest evidence for promoting adherence in patients with immune-mediated inflammatory disorders [40].

**Table 4:** Step-wise approach to identify RRMS patients with a high risk (>50%) of early injectable DMD treatment discontinuation and to guide persistence improving measures

	To be assessed	Tool	Outcome	Action
Step 1	Physical HRQoL Control self-efficacy	MQoL-54 MSSES	Predictive score	If score in lowest quartile (>50% discontinuation risk) → 2A & 2B
Step 2A	Depression & anxiety Fatigue Bladder	HADS MFIS Actionable	Diagnostic scores	If score(s) abnormal → further diagnosis and therapy If MSSES score(s) low → therapy?
Step 2B	Prognosis	1) Clinical 2) MRI 3) CSF	Prognostic indicators	If prognosis unfavourable > consider 2^nd^line DMD

*MS* multiple sclerosis, *DMD* disease modifying drug, *HRQoL* health-related quality of life, *MSQoL*-54 Multiple Sclerosis Quality of Life-54, *MSSES* Multiple Sclerosis Self-Efficacy Scale, *HADS* Hospital Anxiety and Depression Scale, *MFIS* Modified Fatigue Impact Scale, *MRI* magnetic resonance imaging, *CSF* cerebrospinal fluid

Importantly, the approach that we propose does not depend on doctor- or nurse-based scores, and therefore its interference with daily neurological practice will be limited. Steps 1 and 2a are based on patient-reported outcomes, preferentially obtained via web-based questionnaires that are completed at home [41]; this contrasts with e.g. the time-consuming Expanded Disability Status Scale score. Ideally, patients complete the HRQoL and self-efficacy questionnaires prior to the outpatient consultation; the predictive score is generated automatically via an algorithm; online presented to the patient, neurologist and nurse [41, 42]; and, if indicated, questionnaires for additional assessments are made available. In our opinion, the use of web-based patient-reported outcomes and their integration in the daily care process, makes persistence-promoting solutions feasible at the individual and practice level [43].

As to the limitations of our study, it may be argued, first, that the predictive score is less applicable to the INFb group of injectable DMDs, as these may negatively affect fatigue and mood, both major constituents of HRQoL. However, as it is the baseline value of HRQoL that contributes to the prediction of persistence, and not the HRQoL during treatment, such an effect may be questioned. Moreover, in a previous study in IM INFb-treated patients we found that lower physical or mental HRQoL at baseline was indeed associated with treatment discontinuation within 24 months [13]. Second, the predictive score is based on a single data set that was obtained in patients treated with the same injectable DMD. Therefore, the predictive value of the score should be confirmed in prospective studies and in patients treated with other DMDs.

## Conclusions

Findings in this prospective web-based patient-centred study in RRMS patients starting treatment with GA 20 mg sc daily suggest that pre-treatment physical HRQoL and control self-efficacy may identify patients with a high risk of early injectable DMD treatment discontinuation. Based on these results we propose a step-wise approach to identify high-risk patients and to efficiently choose individualized persistence-promoting measures in daily practice. Future studies are needed to validate the predictive model and to assess the (cost-) effectiveness of the proposed follow-up.

## Additional files

Additional file 1: Data on adherence, persistence, self-efficacy, HRQoL, gender and disease duration. (XLSX 25 kb)
Additional file 2: Data on logistic regression analysis. (DOC 56 kb)

## Abbreviations

AJAX: Asynchronous JavaScript and XML
CAIR: Correlative analysis of adherence in relapsing remitting multiple sclerosis
DMD: Disease modifying drug
eCRF: Electronic case record form
GA: Glatiramer acetate
HRQoL: Health-related quality of life
INFb: Interferon-beta
MS: Multiple sclerosis
MSQoL-54: Multiple sclerosis quality of life-54 items
MSSES: Multiple sclerosis self-efficacy scale
RRMS: Relapsing remitting multiple sclerosis
SD: Standard deviation
VPN: Virtual private network
XML: Extensible markup language
