# Supplementary material for: Glatiramer acetate treatment persistence - but not adherence - in multiple sclerosis patients is predicted by health-related quality of life and self-efficacy: a prospective web-based patient-centred study (CAIR study)
Source: Health Qual Life Outcomes. 2017 Mar 14;15:50. doi: 10.1186/s12955-017-0622-z (PMC5351176; doi:10.1186/s12955-017-0622-z)
Supplement: Additional file 2: — Data on logistic regression analysis. (DOC 56 kb) [file 12955_2017_622_MOESM2_ESM.doc]

CAIR study: (databank 15 mei 2012) PAT(203), met ZVL(115) (P.J.Jongen)

=========== tav artikel completering januari 2016

PUNT.1 P(kans = stoppen): uit diverse basale algmene parameters

Logistic voorspel P(kans = stoppen) uit meerdere basale parameters

Nu logistische regressie met ALLEEN MS54SC(phy/men) en MSSES(F/C) maand 0

| The CONTENTS Procedure |
| --- |

| Data Set Name | WL.PJ_CAIR2BASIS | Observations | 200 |
| --- | --- | --- | --- |
| Member Type | DATA | Variables | 12 |
| Engine | V9 | Indexes | 0 |
| Created | maandag 09 mei 2016 08:40:41 uur | Observation Length | 96 |
| Last Modified | maandag 09 mei 2016 08:40:41 uur | Deleted Observations | 0 |
| Protection |  | Compressed | NO |
| Data Set Type |  | Sorted | NO |
| Label |  |  |  |
| Data Representation | WINDOWS_32 |  |  |
| Encoding | wlatin1 Western (Windows) |  |  |

| Engine/Host Dependent Information | |
| --- | --- |
| Data Set Page Size | 8192 |
| Number of Data Set Pages | 3 |
| First Data Page | 1 |
| Max Obs per Page | 84 |
| Obs in First Data Page | 57 |
| Number of Data Set Repairs | 0 |
| Filename | h:\Biostatistiek\Projecten\Projecten WL\2011\2011_sjef_jongen\cair_studie_april2012\sasdatafiles\pj_cair2basis.sas7bdat |
| Release Created | 9.0202M3 |
| Host Created | XP_PRO |

| Variables in Creation Order | | | | | |
| --- | --- | --- | --- | --- | --- |
| # | Variable | Type | Len | Format | Label |
| 1 | Patient | Num | 6 |  | Patient id |
| 2 | adherence95 | Num | 8 | ADF. | Drug adherence (percentage > 95 = 1) |
| 3 | compl_eos | Num | 8 | NJF. | Completion of the Study(0-no, 1-yes) |
| 4 | msses_m0_f | Num | 8 | 8.2 | MSSES-maand-0 Functie (gem. F1 t/m F9) |
| 5 | msses_m0_c | Num | 8 | 8.2 | MSSES-maand-0 Controle (gem. C1 t/m C9) |
| 6 | ms54sc_phy_m0 | Num | 8 |  | MS54 Physical Health composite Maand-0 |
| 7 | ms54sc_men_m0 | Num | 8 |  | MS54 Mental Health composite Maand-0 |
| 8 | gesl | Num | 8 |  | Geslacht: (0=vrouw, 1=man) |
| 9 | v4938_j | Num | 8 |  | Disease duration (in yrs) |
| 10 | disease_duur | Num | 8 |  | Disease duration: (0: <= 2 jaar, 1: > 2 jaar) |
| 11 | p_mod_stop | Num | 8 |  | Estimated Probability |
| 12 | pstop_kl | Num | 8 |  | estimated prob. (quartile class) |

>> Program in: 2016\cair_pj_anal10_def_160509.sas SAS-process[09MAY16, 08:40] <<
